# Network neighbors of viral targets and differentially expressed genes in COVID-19 are drug target candidates

**DOI:** 10.1038/s41598-021-98289-x

**Published:** 2021-09-23

**Authors:** Carme Zambrana, Alexandros Xenos, René Böttcher, Noël Malod-Dognin, Nataša Pržulj

**Affiliations:** 1 Barcelona Supercomputing Center, Barcelona, Spain; 2 Department of Computer Science, University College London, London, WC1E 6BT UK; 3 ICREA, Pg. Lluís Companys 23, Barcelona, Spain

**Keywords:** Computational biology and bioinformatics, Data integration, Data mining, Network topology

## Abstract

The COVID-19 pandemic is raging. It revealed the importance of rapid scientific advancement towards understanding and treating new diseases. To address this challenge, we adapt an explainable artificial intelligence algorithm for data fusion and utilize it on new omics data on viral–host interactions, human protein interactions, and drugs to better understand SARS-CoV-2 infection mechanisms and predict new drug–target interactions for COVID-19. We discover that in the human interactome, the human proteins targeted by SARS-CoV-2 proteins and the genes that are differentially expressed after the infection have common neighbors central in the interactome that may be key to the disease mechanisms. We uncover 185 new drug–target interactions targeting 49 of these key genes and suggest re-purposing of 149 FDA-approved drugs, including drugs targeting VEGF and nitric oxide signaling, whose pathways coincide with the observed COVID-19 symptoms. Our integrative methodology is universal and can enable insight into this and other serious diseases.

## Introduction

The ongoing COVID-19 pandemic exposed the shortcomings of healthcare systems and devastated the economy^1–3^. A major issue is the lack of adequate medications. This has mostly been addressed by extrapolating drug targets from related viruses and assessing the efficacy of approved drugs^4–7^. Once an effective vaccine has been developed, immunizing most of the population will pose serious other challenges, including economic and logistic ones. Thus, treatment options for patients is a key issue that will remain relevant.

The COVID-19 disease is caused by a betacoronavirus termed severe acute respiratory syndrome coronavirus 2 (SARS-CoV-2). This virus reproduces in the upper respiratory tract and is highly infectious due to asymptomatic carrier transmission^8,9^. As a (+)RNA virus, SARS-CoV-2 completely depends on infected host cells to replicate and thus, interactions with the host molecular network are crucial in avoiding the host immune response and reprogramming the cell to enforce its reproduction^10^. SARS-CoV-2 binds to a cellular receptor to enter a host cell, the exopeptidase angiotensin converting enzyme 2 (ACE2)^11^. Upon ACE2 binding, transmembrane protease, serine 2 (TMPRSS2), is required to prime the viral spike protein and allow the virus to enter the host cell via endocytosis^12,13^. Once a cell has been infected, the synthesized viral proteins can interact with a number of host factors to perform viral functions, likely by modulating cellular processes ranging from vesicle trafficking to regulating gene-expression and ubiquitination^5^. An inflammatory response to the SARS-CoV-2 infection had been revealed by 1910 differentially expressed host genes (DEGs) in infected lung tissue^14^. Elevated glucose levels and glycolysis have been shown to promote SARS-CoV-2 replication and cytokine production in monocytes^15^. Thus, targeting metabolic pathways may provide new strategies to treat COVID-19 disease. While many studies focused on ACE2, TMPRSS2 and other direct viral interaction targets as candidates for treating SARS-CoV-2^12,16,17^, few studies have investigated the positioning of the protein targets in the host molecular interactome and the possible impacts of such positioning^18,19^. Interestingly, the DEGs from Blanco-Melo et al.^14^ show little overlap (1.78%) with human proteins that directly interact with the viral ones (described by Gordon et al.^5^). Thus, the underlying molecular mechanisms, from the proteins targeted by the virus to the ones altered once the infection is onset, is not fully understood.

Novel insights have been found by integrating several different molecular interaction network types, by using data fusion algorithms, such as finding cellular wiring patterns specific to disease (“rewired genes” in disease compared to control), that can also be used for predicting new cancer-related genes^20,21^. These data fusion algorithms are based on Non-Negative Matrix Tri-Factorization (NMTF), which approximates a high-dimensional data matrix that contains relations between two entities (e.g. between proteins and drugs) as a product of three low-dimensional, non-negative matrices (called factors)^22^. NMTF based methods were initially proposed for dimensionality reduction and co-clustering due to its relatedness to k-means clustering^22–24^. The clustering information is encoded in the low-dimensional matrix factors, named cluster indicator matrices. Moreover, NMTF is also an intermediate data integration method, by which several relational matrices can be decomposed simultaneously sharing a matrix factor across the decompositions, that directly integrates all datasets through the inference of a single joint model; it can also be used for predicting new entries in the input matrices due to the matrix completion property^20,21,25,26^. Unlike other artificial intelligence algorithms, this method is interpretable and its predicted values are traceable, which are essential properties when mining biological data. These inherent features of NMTF, interpretability, dimensionality reduction, co-clustering and prediction of new entries (via matrix completion), have thus far been used for predicting disease associations^25^, protein–protein interactions^23^ and gene functions^27^, as well as for discovering disease-related genes^28^. Moreover, for cancer, this data fusion framework successfully uncovered patient subgroups with different prognostic survival outcomes, predicted novel cancer-related genes and proposed drugs for re-purposing^21^. Thus, to predict candidate target genes and the existing drugs that could be re-purposed for treating COVID-19, we adapt our versatile data fusion framework to fuse viral host interactions, human protein interactions, and drug data^21^. Among the predicted drug-target interactions (DTIs), we observed that one-third of the targeted genes directly connect the host proteins targeted by the viral proteins (we termed them viral interactors (VIs)) and the host differentially expressed genes (DEGs). Thus, we decided to further explore how the VIs and the DEGs are connected in the host interactome.

The host molecular interactome is usually modeled by using networks, where biological entities (genes, or equivalently in this study, proteins, as gene products) and the interactions between them are represented as network nodes and edges (links), respectively. Networks are widely applicable and are frequently used for representing: physical interactions of proteins via protein–protein interaction (PPI) networks; metabolic interactions (MI) that correspond to known metabolic pathways; or functional associations between genes, such as epistasis via genetic interaction (GI) networks. To obtain information about the organization of a network and the wiring patterns of its nodes, various network properties are being used, ranging from the basic node degree (the number of edges incident to the node; the higher the degree, the more “degree central” the node) to several other measures of network centrality^29^. Furthermore, local and global network topology can be assessed by using graphlets, small, connected, non-isomorphic, induced subgraphs of large networks, that provide a quantitative measure of the wiring pattern around a node in the network and thus have been used for various applications, including for node centrality and network distance measures^30,31^. In particular, Graphlet Degree Vectors (GDVs)^30^ capture the local wiring patterns for each node in a network. Studying the topology (structure) of molecular interaction networks revealed that genes (or proteins) with similar biological functions are either neighbors in the network that tend to form clusters, or are characterized by similar wiring patterns, independent of being neighbors in the network^30,32^. Thus, to investigate the interplay between the human proteins that are viral interactors (VI) and those human genes (or equivalently proteins) that are differentially expressed after the infection (DEGs), we study these two protein sets and their neighbors in the context of the human interactome. We use a holistic view of the human interactome by merging the PPI, GI and MI networks in the molecular interaction network (MIN). We find that the neighbors in the human MIN of these two sets have a large overlap (we term the genes in the overlap the “common neighbors”) containing central genes (with larger node degree in the MIN). Moreover, we find that they are enriched in viral processes, and hence, they might be involved in the COVID-19 mechanisms.

We find new drug–target interactions that open new ways for potential COVID-19 treatments. Firstly, we predict candidate target genes and the existing drugs that could be re-purposed for treating COVID-19 by disrupting the disease mechanisms. Moreover, we observe that one third of the targeted genes directly connect the viral interactors (VIs) and the host differentially expressed genes (DEGs). Secondly, we uncover that in the human interactome VIs and DEGs, while mostly disjoint, are indirectly connected by their neighbors (common neighbor genes). Furthermore, we find that the common neighbor genes might be key to the infection mechanisms used by the virus since they are enriched in various viral processes. Finally, we investigate the biological mechanisms that the predicted candidate target genes are involved in and their relevance for treating COVID-19. We discover that the targeted genes participate in two molecular pathways, nitric oxide and VEGF signaling, whose functions strongly correlate with several observed COVID-19 symptoms.

## Results

**Figure 1:**
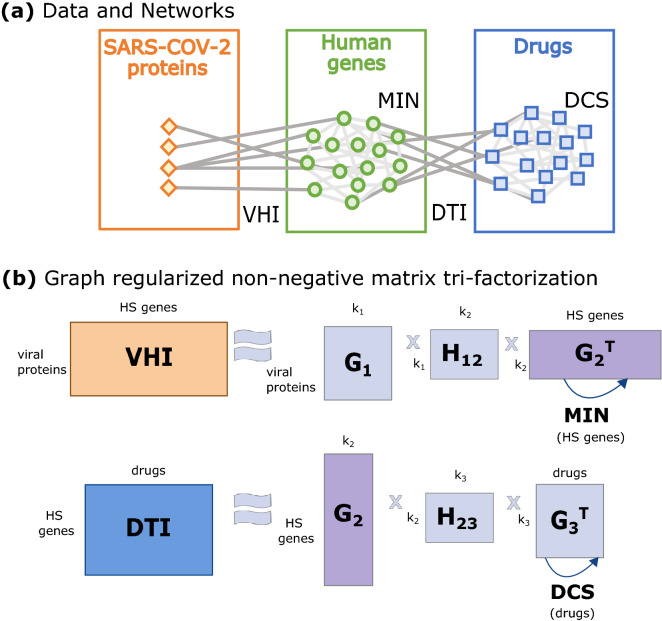
Illustration of the data and framework. **(a)** Schematic illustration of datasets used in this study. Three data types are represented: SARS-CoV-2 proteins (in orange), human genes (in green) and drugs (in blue). Two relational datasets connect different types of data: virus-host protein–protein interactions (VHIs) and drug-target interactions (DTIs). Network structural knowledge from these data types is contained in the molecular interaction network (MIN) and the drug chemical similarity (DCS) network. **(b)** Graph-regularized non-negative matrix tri-factorization (GNMTF) used for fusing the VHIs, DTIs, MIN and DCS networks. The matrix factor $G_2$ is shared across decompositions to simultaneously decompose the VHI and DTI networks. Network structure (topology) information from the MIN and DCS networks are incorporated into the data fusion by using two regularization terms (illustrated by arcs with arrows). The parameters $k_1$, $k_2$ and $k_3$ indicate the numbers of clusters of viral proteins, human genes and drugs, respectively.

We adapt our data fusion framework based on graph-regularized non-negative matrix tri-factorization (GNMTF) to fuse two heterogeneous networks, viral-host interactions (VHIs) and previously known drug-target interactions (DTIs), containing three different data types: SARS-CoV-2 proteins, human genes and drugs (either FDA-approved and experimental). To add information of the relation of the human genes, we use a holistic view of the human interactome by merging the PPI, GI and MI networks in the molecular interaction network (MIN). To add the relation among drugs, we used the Drug Chemical Similarity (DCS) network (Fig. 1; for more details on the data that we used see “Datasets, pre-processing and matrix construction” section and on our framework see “Data fusion framework tailored to SARS-CoV-2” section in “Methods” section).

To have a holistic view of the relationships between genes, we created the MIN network by merging PPI, GI and MI networks. In particular, we add the MI network, since it has been demonstrated that metabolic processes, such as glycolysis, promote SARS-CoV-2 replication, and hence, targeting metabolic pathways might be key for treating COVID-19^15^ (for more details, see section “Datasets, pre-processing and matrix construction” in “Methods” section). Note that we validate that the topology (structure) of the PPI network dominates the MIN by comparing commonly used network properties and the wiring patterns of the constituent networks and the MIN (for more details, see “Comparison of the molecular interaction network and its constituent networks” section in Supplementary Materials).

### The data fusion framework predicts novel DTIs for SARS-CoV-2

Before using our framework to predict novel DTIs for COVID-19, we first validate that it captures the functional relationships between genes (as captured by Gene Ontology (GO) annotations) and between the drugs (as captured by DrugBank “Drug Category” (DC) annotations). We assess the capability of the framework to predict new DTIs by using tenfold cross-validation and we also validate $32.64\%$ of the newly predicted DTIs through external databases. Finally, we find that one third of the targeted proteins from the predicted DTIs directly connect the host proteins that interact with the viral proteins and the host proteins coded by the differentially expressed genes in COVID-19 infection.

To assess that the framework captures the functional relationships between genes (as captured by Gene Ontology (GO) annotations) and between the drugs (as captured by DrugBank “Drug Category” (DC) annotations), we perform an enrichment analysis on the gene and drug clusters obtained by the framework (for more details, see “Extracting clusters of genes and drugs” section in “Methods” section). The enrichments of both the gene and the drug clusters are statistically significant and more than $80\%$ of the clusters show enrichments (for more details, see “The data fusion framework preserves the biological relations between genes and drugs” section in Supplementary Materials). Hence, the joint decomposition of VHIs and DTIs successfully extracts functional information about genes and drugs, respectively.

**Figure 2:**
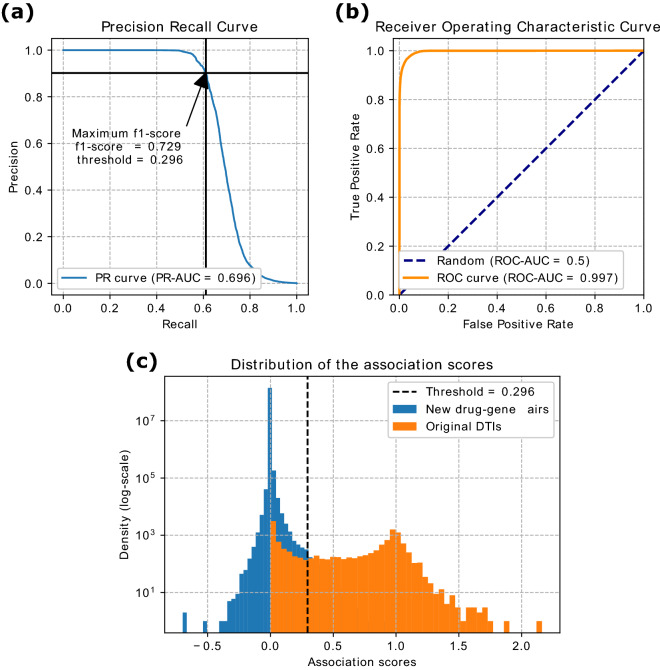
Prediction of new DTIs. **(a)** Precision-Recall (PR) and **(b)** Receiver Operating Characteristic (ROC) curves for assessing the ability of the framework to capture the known drug–target interactions accurately. *AUC* area under the curve. **(c)** Distribution of the association scores of the reconstructed matrix, for the input DTIs (orange) and the new drug–gene pairs obtained by the matrix completion property of GNMTF (blue). New drug–gene pairs on the right side of the threshold (dashed line) were considered to be newly predicted DTIs.

To predict new DTIs, we used the matrix completion property to reconstruct the DTI matrix. Each entry of the reconstructed matrix contains an association score, $s_A$, corresponding to a drug–gene pair. This score can be interpreted as a relative measure of confidence for each drug–gene association (for more details, see “Prediction of new drug–target interactions for drug re-purposing” section in “Methods” section). Then, we assess if score $s_A$ can be used to separate DTIs from non-interacting gene-drug pairs performing precision-recall (PR) and receiver operating characteristic (ROC) curves analysis using all the input DTIs as ground truth. As illustrated in Fig. 2a,b, the corresponding scores are $PR\text {-}AUC=0.696$ and $ROC\text {-}AUC=0.997$ (for more details, see “Prediction of new drug–target interactions for drug re-purposing” section in “Methods” section). In addition, we showed that $s_A$ score can predict unseen DTIs by using 10-fold cross-validation, resulting in PR-AUC=$0.332 \pm 0.014$ and ROC-AUC=$0.847 \pm 0.014$ over the validation set (mean and standard deviation with respect to the 10 folds; for more details, see “The data fusion framework can predict unseen DTIs” section in Supplementary Materials). Finally, to predict new DTIs, we define an optimal threshold based on $s_A$ using F1-score and then, we consider a false positive as predicted DTIs. The best F1-score ($F_1 = 0.729$) is associated with a threshold of $s_A = 0.296$, yielding 814 newly predicted DTIs with 565 (FDA-approved and experimental) drugs targeting 172 genes (Fig. 2c, Supplementary Table S2). Moreover, we showed that the framework uncovers additional DTIs when using the MIN compared to using the PPI network. In particular, 93.8% of the 533 DTIs predicted using the PPI network are also predicted using the MIN, but using the MIN the framework also uncovers 38.6% (314) additional DTIs that could not be predicted when using the PPI network (for more details, see “The holistic view of the human interactome uncovers additional DTIs” section in Supplementary Materials). Due to the urgent need for finding a treatment for COVID-19, we focus on those DTIs that include only FDA-approved drugs, yielding 573 newly predicted DTIs with 369 drugs targeting 143 genes. We find that 187 out of the 573 predicted DTI (32.64%) were present in four external databases, namely Drug Central, Comparative Toxicogenomics Database, PharmaGKB and Therapeutic Targets Database (Supplementary Table S2). The DTIs present in the four external databases were not used as input into our fusion framework, and hence we can use them to validate the newly predicted DTIs.

Interestingly, among the 143 genes targeted in the predicted DTIs obtained by our data fusion only one is a host protein targeted by the viral proteins; it is HDAC2 targeted by cannabidiol. To explore the other 142 genes and their possible relations with SARS-CoV-2 infection, we study their connection to the host proteins that interact with the viral proteins (we termed them viral interactors (VIs)) in the context of the MIN. We find that 58 drug targeted genes obtained by the data fusion are direct neighbors of the VIs and the remaining 84 genes are at distance 2 or 3 in the MIN from the VIs (79 are at distance 2 and 5 are at distance 3). In addition, to further explore the relation of the genes targeted by COVID-19 proteins after the infection, we study the connection of the drug targeted genes obtained by our data fusion with the differentially expressed genes (DEGs) in COVID-19 infection described by Blanco-Melo et al.^14^ in the context of MIN. We find that 10 out of the 143 drug targeted genes obtained by our data fusion are DEGs, 100 out of the 143 genes are neighbors of the DEGs and the rest (33 out of the 143) are at distance 2 from the DEGs in COVID-19 infection. Furthermore, we find that 49 out of the 143 genes are at the same time neighbors of the VIs and DEGs. These genes that connect the VIs and DEGs might be key targets for disrupting the disease mechanisms.

In summary, we successfully predict new DTIs between the human targets and existing drugs that could be re-purposed. Moreover, we assess through external databases one third of the predicted DTIs. Lastly, when focusing on the targeted proteins in the predicted DTIs, we find that one third of the targeted proteins directly connect the host proteins that interact with the viral proteins and the host proteins coded by the differentially expressed genes in COVID-19 infection (i.e. they are neighbors of both), hence indicating that our predicted DTIs may hit the human interactome at the points that can disrupt the viral mechanisms going from the binding of the SARS-CoV-2 viral protein to the host protein towards the differentially expressed host gene in COVID-19 infection (detailed below).

### Topological analysis of the human interactome reveals key genes for explaining the molecular mechanisms of SARS-CoV-2

After finding that, in the MIN, one third of the human targets in the predicted DTIs directly connect the human proteins that interact with the viral proteins (viral interactors, VIs) and those corresponding to differentially expressed genes (DEGs) in COVID-19 infection, we further explore all the genes that connect the VIs and the DEGs in the human interactome (we termed them common neighbors), in particular in the above described MIN. Our reasoning is that neighboring genes can act as links between the signal inputs, VIs, and the observed outputs, such as dysregulated genes, and may thereby be involved in the disease mechanisms. In particular, we show that the common neighbor genes are central in the MIN, we assess the similarity in biological functions between the common neighbor and VIs genes by comparing their wiring patters and we demonstrate that the biological functions of the common neighbor genes are related to viral processes.

**Figure 3:**
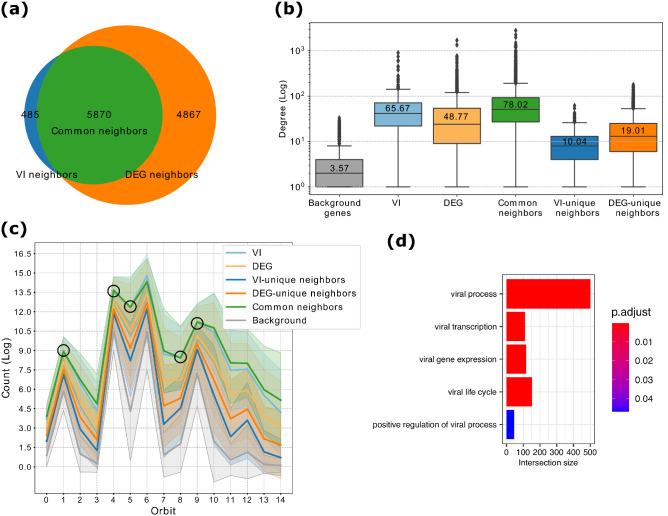
Common Neighbors are key genes in the molecular interaction network (MIN). **(a)** Venn Diagram showing the overlap between VI neighbor and DEG neighbor gene sets. **(b)** Comparison of the average degrees (given on the vertical axis) for the different gene sets (given on the horizontal axis) based on their degrees in the MIN. All pair-wise comparisons between these are statistically significant with p < 0.0001, using two-sided Mann–Whitney–Wilcoxon test. **(c)** GDV signatures for the VI, DEG, VI-unique neighbors, DEG-unique neighbors, common neighbors and background gene sets. All pair-wise comparisons between the counts (on the vertical axis) of the orbits (denoted by 0 to 14 on on the horizontal axis) of common neighbors and the rest of the gene sets are statistically significant with p < 0.05, using two-sided Mann-Whitney-Wilcoxon test, except for the orbits marked with a black circle between common neighbors and VI gene set. **(d)** Enrichment analysis of the common neighbor genes in the MIN. The bar plot is showing the enrichments related to viral processes. The complete list can be found in Supplementary Table S5. The intersection size (on horizontal axis) is the number of common neighbors genes that are annotated with the corresponding GO term (on the vertical axis). The adjusted p-value, “p.adjust”, is obtained by the *Set Counts and Sizes* correction method. This method considers the dependency of multiple tests by taking into account the overlap of functional terms (on colorbar).

We use the 332 host genes reported by Gordon et al.^5^ as the set corresponding of viral interactors (we term this gene set the “VI”). For the DEG set, we use the 1,910 DEGs identified by Blanco-Melo et al.^14^ in lung tissue samples from 2 infected patients (see “Datasets, pre-processing and matrix construction” section in “Methods” section). Furthermore, since previous studies showed that disease genes tend to form densely connected communities^33^ in the MIN, we identify direct network neighbors of both of the above described gene sets (we term these two new gene sets the “VI neighbors” and “DEG neighbors”). As shown in Fig. 3a, these two sets have $52.30\%$ of overlap (statistically significant with p-value $< 1e^{-16}$ (the exact p-value is not provided due to the fact that p-values in Python are float64 objects (i.e. 16 decimals are reported) and very small p-values are rendered to 0), using hypergeometric test; for more details, see “Analysis of the molecular interaction network and its wiring patterns” section in “Methods” section) and hence, we also explore this overlap as a separated gene set (termed the “common neighbors”). Thus, VI and DEG genes, while mostly disjoint, are largely ($52.30\%$) indirectly connected by their neighbors. To fully explore the entire set of neighbors in the MIN network of proteins participating in VIs and the protein products of DEGs in COVID-19 disease, we study separately those VI neighbor and DEG neighbor genes that overlap and those that do not overlap, and within those that do not overlap, we term the neighbors of only VIs the “VI-unique neighbors” and the neighbors of only DEGs the “DEG-unique neighbors”. The rest of the genes in the MIN that are not present in any of these five gene sets (VI, DEGs, VI-unique neighbors, DEG-unique neighbors, common neighbors) are term “background genes”.

To establish whether a SARS-CoV-2 infection affects proteins that are central in the MIN, we analyze the above described gene sets by the following commonly used network properties: four centrality measures (degree, eigenvector, betweenness and closeness centralities) and the clustering coefficient (for more details, see “Analysis of the molecular interaction network and its wiring patterns” in “Methods” section). As shown in Fig. 3b, VI and DEG genes show significantly higher degree centralities (p < 0.0001) compared to the background genes, indicating their importance in the MIN. In addition, genes in both of these sets have a higher clustering coefficient than the background genes, indicating their higher tendency to form clusters (Table 1). Notably, the common neighbor gene set exceeds both VI and DEG genes in all of these measures except for closeness centrality. Thus, common neighbor genes are likely to participate in many functions, since they are central in the MIN. The VI-unique and DEG-unique neighbor genes have lower centralities compared to the VI, DEG and common neighbor genes, which confirms the relevance of the common neighbors followed by the VI-unique and DEG-unique neighbor genes. Therefore, common neighbor genes are highly connected and central genes that, in particular, connect the proteins targeted by the virus to the ones deregulated after the infection, and hence, they might be key for understanding the underlying molecular mechanism of COVID-19.

**Table 1:** Network properties of molecular interaction network (MIN), focusing on the following gene sets: viral interactors (VI), differentially expressed genes after infection (DEG), overlap of the direct network neighbors in the MIN of these two sets (common neighbors), neighbors of the VI and DEG gene set that were not in the common neighbor genes set (VI-unique neighbors and DEG-unique neighbors), and the rest of the genes in the MIN (background genes). The common neighbor gene set exceeds the other genes sets in all of the measures except for closeness centrality, in which VI gene set has the highest value (the highest value for each measure is highlighted in bold).

	Average degree	Eigenvector centrality	Clustering coefficient	Betweeness centrality	Closeness centrality
VI	65.67	0.006282	0.137887	0.000194	**0.359875**
DEG	48.77	0.004282	0.14323	0.000168	0.340381
Common neighbors	**78.02**	**0.006764**	**0.186346**	**0.00027**	0.358132
VI-unique neighbors	10.04	0.00095	0.156097	0.000009	0.318445
DEG-unique neighbors	19.01	0.001446	0.152142	0.000028	0.326536
Background	3.57	0.000291	0.096368	0.000003	0.293636

To assess whether the genes participating in the aforementioned sets have similar biological functions in the MIN network, we compare their wiring patters, by using their Graphlet Degree Vectors (GDVs)^30^ (for more details, see “Analysis of the molecular interaction network and its wiring patterns” in “Methods” section). Previous molecular networks analyses revealed that genes with similar biological functions tend to group together and have similar wiring patterns in molecular networks^34^. As shown in Fig. 3c, GDV of the common neighbor genes is different from the GDVs of the rest of the gene sets, except for the GDV of the VI. We verify this by computing the Mann-Whitney U test (with a significance level of 0.05) for each pair of orbits (Supplementary Table S4). Only five orbit counts are not statistically significantly different between the common neighbor genes and the VIs. Namely the orbits 1, 4, 5, 8 and 9 (Fig. 3c orbits marked with a circle; Supplementary Table S4 marked in bold). Thus, the common neighbor genes have different wiring patterns compared to the other gene sets, and only show some similarities with the wiring patterns of VIs genes. This indicates that the common neighbors might have similar biological functions that could be related to SARS-CoV-2 infection.

To investigate whether the biological functions of the common neighbor genes in the MIN are related to COVID-19, we perform a functional enrichment analysis across multiple functional annotation databases: Gene Ontology (GO), KEGG, REACTOME and CORUM (for more details, see “Enrichment analysis of gene and drug clusters” in “Methods” section). Among the significantly enriched terms, many are related to viral infection processes (for the full list of enriched terms, see Supplementary Table S5). As shown in Fig. 3d, the enriched GO terms related to viral infection processes have a large intersection size (i.e., the number of common neighbor genes that are annotated with the corresponding GO term). In particular the general viral process term annotates almost 500 common neighbor genes. We perform the same enrichment analysis for the rest of the gene sets and find that VI-unique neighbor, DEG-unique neighbor and background genes are not enriched in viral processes (see Supplementary Tables S6–S8). These results indicate that the common neighbor genes participate in SARS-CoV-2 infection and hence, they might be potential drug targets to treat COVID-19.

Based on these results, we conclude that SARS-CoV-2 proteins mainly interact with central human proteins, or influence the expression of host proteins that are central in the MIN. Moreover, we find that the neighbors of these two gene sets (common neighbor genes of the VIs and the DEGs) are also central in the MIN. Interestingly, the common neighbor genes are enriched in viral related processes, while the VI-unique neighbor, DEG-unique neighbor and background genes are not. Thus, these common neighbor genes (listed Supplementary Table S9) are likely to be involved in COVID-19 disease and they might be key for explaining the mechanisms that go from the host proteins targeted by the viral proteins to the differentially expressed genes resulting from the COVID-19 infection.

### Predicted DTIs involving FDA-approved drugs targeting common neighbor genes disrupt biological mechanisms relevant for COVID-19

After discovering that the common neighbor genes (those that directly connect the host proteins that interact with the viral proteins and the proteins corresponding to differentially expressed genes in COVID-19 infection) are likely to be important in SARS-CoV-2 infection, we focus on the predicted DTIs that target these common neighbor genes; we term these DTIs “common neighbor DTIs”. The common neighbor DTIs contain 185 DTIs targeting 49 common neighbor genes with 149 drugs (see Supplementary Table S10). First, we investigate how many of the 149 drugs targeting the common neighbors are currently studied in COVID-19 context. Then, to investigate which biological mechanisms are targeted by the common neighbor DTIs, we perform a functional enrichment analysis of the 49 genes targeted in these DTIs. Finally, we manually check the enriched pathways and discuss their relevance in the context of COVID-19.

We check whether any of these 149 drugs targeting common neighbor genes have been investigated for treating COVID-19; we use the CORona Drug InTEractions (CORDITE) database (https://cordite.mathematik.uni-marburg.de). Also, we ask whether they are part of interventional clinical trials currently being conducted (retrieved from https://clinicaltrials.gov). As shown in Supplementary Table S10, $17.44\%$ and $11.40\%$ of the drugs involved in the common neighbor DTIs are listed in CORDITE and subject to at least one active clinical trial on COVID-19, respectively. These results demonstrate the relevance of the predicted DTIs.

**Figure 4:**
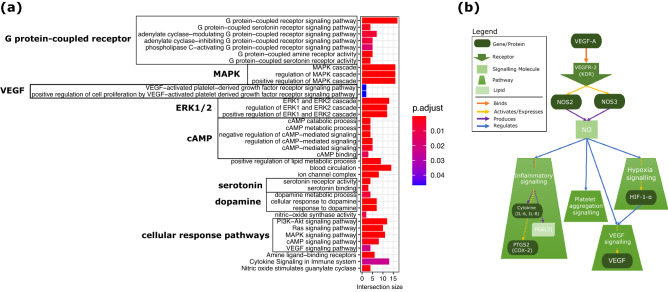
Biological mechanisms targeted by the common neighbor DTIs. **(a)** Enrichment analysis of the targets in our “common neighbor DTIs”. The bar plot is showing the enrichments related to G protein-coupled receptors; MAPK cascade; VEGF and ERK1/2; cAMP/cGMP signaling; lipid metabolism and blood circulation; ion channel activity and response to amine ligand-binding, particularly serotonin and dopamine; cellular response pathways and terms linked with amine ligand-binding receptors, cytokine and nitric oxide (NO) signaling. The complete list can be found in Supplementary Table S11. The intersection size (on horizontal axis) is the number of genes in our “common neighbor DTIs” that are annotated with the corresponding GO term or pathway from KEGG or REACTOME (on the vertical axis). The adjusted p-value, “p.adjust”, is obtained by the *Set Counts and Sizes* correction method. This method considers the dependency of multiple tests by taking into account the overlap of functional terms (on colorbar). **(b)** Illustration of how the enriched pathways are tied to NO and VEGF signaling, showing that NO production is directly related to VEGFR-2 receptor and at the same time NO regulates VEGF signaling pathway among others: inflammatory signaling, hypoxia signaling and platelet aggregation.

We perform an enrichment analysis across multiple functional annotation databases: Gene Ontology (GO), KEGG, REACTOME and CORUM (for more details, see “Enrichment analysis of gene and drug clusters” section in “Methods” section). As illustrated in Fig. 4a, the 49 genes involved in the common neighbor DTIs are enriched in several GO terms in all three GO domains (i.e. Biological Process, Cellular Component, Molecular Function). Namely, they are terms related to: G protein-coupled receptors; tyrosine kinase-mediated activation of MAPK signaling, in particular VEGF and ERK1/2; cAMP/cGMP signaling; lipid metabolism and blood circulation; ion channel activity and response to amine ligand-binding, particularly serotonin and dopamine. Likewise, when testing for the enrichment of KEGG and REACTOME pathway terms, we find enrichments of cellular response pathways (PI3K-AKT, Ras, MAPK, cAMP, VEGF) and terms linked with amine ligand-binding receptors, cytokine and nitric oxide (NO) signaling (the complete list of enriched terms can be found in Supplementary Table S11).

Upon closer inspection, many of these pathways are either directly or indirectly tied to NO and VEGF signaling, which are also connected to each other (see Fig. 4b). For instance, KDR (VEGFR-2) is required for VEGF-A mediated induction of NOS2 and NOS3, leading to the production of the signaling molecule NO by macrophages (NOS2) and endothelial cells (NOS3)^35^. Increased NO also directly affects inflammatory signaling by regulating cytokine (IL-6, IL-8) and PGE(2) production^36,37^ as well as PTGS2 (COX-2) activation^38^. It is recognized as a key regulator of both VEGF synthesis and platelet aggregation^39,40^. Lastly, NO is also tied to hypoxia signaling by direct interaction with key components such as HIF-1-alpha, which in turn regulates VEGF signaling^41,42^.

Notably, striking similarities between these NO and VEGF signaling-related functions and COVID-19 symptoms can be observed. Vascular complications are common in COVID-19 patients^43^. In particular, recent studies on COVID-19 patients have reported an increased in VEGF levels and platelet activity, as well as extensive blood clotting and endothelial injury as a sign of direct infection of endothelial cells^44–47^. Moreover, cytokine storms and IL-6 have been related to severe disease COVID-19^48,49^, with macrophages being potential key players^50^. Finally, neurological symptoms have also been recognized in COVID-19 patients, and hypoxic injury is one of the possible explanations for the observed tissue damage^51,52^.

NO signaling might be central in understanding the disease, since the anatomic sites of COVID-19 symptoms, lung, heart, circulatory system and brain, also correlate with the expression patterns found for the three known human NO synthases: NOS1 (neural NOS; expressed in peripheral neurons), NOS2 (endothelial NOS; expressed in endothelial cells, cardiac myocytes, cardiac conduction tissue) and NOS3 (cytokine-inducible NOS; expressed in endothelial cells, myocytes, macrophages)^53^. Therefore, we propose to further investigate the well tolerated drugs that modulate NO signaling and its related pathways. A potential candidate from our list of common neighbor DTIs is triflusal, which is known to interact with NFKB, NOS2, PDE10A as well as PTGS1, and for which we predict PTGS2 and NOS3 as additional target genes. Triflusal is a trifluoromethylated analogue of acetylsalicylic acid, which is not yet under investigation as COVID-19 treatment, unlike acetylsalicylic acid. Of note, both triflusal and acetylsalicylic acid act as anticoagulants and a recent study associated anticoagulation with lower mortality and intubation rates for hospitalized COVID-19 patients, providing further evidence for the validity of our findings^54^.

Related to VEGF-signaling, we suggest as a putative target gene KDR (VEGFR-2), which appears in the common neighbor DTIs targeted by tyrosine kinase inhibitors, such as Imatinib, Dasatinib, Pexidartinib. These drugs are cancer related drugs with high level toxicity, thus they must be reserved for critically ill cases. Finally, another group of candidate genes from the common neighbor DTIs worth mentioning are phosphodiesterases. Phosphodiesterases are responsible for regulating cAMP/cGMP signaling and hence, they have an interplay with both NO and VEGF^55–57^. Our framework predicted that phosphodiesterases (e.g. PDE4D), could be inhibited by xanthine derivatives such as theophylline.

In summary, by focusing on predicted drug-target interactions involving genes located in the common neighborhood of SARS-CoV-2 VIs and DEGs, we propose a list of 185 DTIs (common neighbor DTIs). For the drugs targeting the common neighbor DTIs, we validate that some of them have been investigated in COVID-19 related studies, or are currently in clinical trials for COVID-19 treatment. For the targeted genes in the common neighbor DTIs, we identify functional enrichments related to cardiovascular integrity, stress signaling and inflammation, all of which can be linked to NO and VEGF signaling. Moreover, both the molecular functions of NO signaling and the expression patterns of NO synthases correlate with reported COVID-19 symptoms, making it a principal target for further study and potentially drug intervention. Finally, our predicted DTIs provide a list of FDA-approved drugs that may be used to target genes related to both the VEGF and NO signaling pathways.

## Discussion

In this work, we adapt our GNMTF-based data fusion framework to predicted candidate target genes and existing drugs that could be re-purposed for treating COVID-19. Moreover, we investigate within the human interactome the interplay between the human proteins that are directly targeted by the SARS-CoV-2 proteins and those genes that are differentially expressed after COVID-19 infection. Our study reveals that the host proteins targeted by viral proteins and the differentially expressed genes are indirectly connected by their neighbors (we termed common neighbor genes). Furthermore, we find that the common neighbors are enriched in various viral processes and hence, might be key to the infection mechanisms used by the virus. By focusing on the predicted drug–target interactions involving FDA-approved drugs and targeting the common neighbor genes, we utilize our integrative framework to predict novel drug-target interactions for genes related to the disease-affected pathways. In particular, we find NO and VEGF signaling as potential molecular pathways whose functions are very similar with several observed COVID-19 symptoms.

In this study, we focus on viral-host protein interactions, specifically on the dataset provided by Gordon et al.^5^, the one available at the time of data collection for our study. Recently, other datasets have been published containing new viral-host protein interactions (e.g., Li et al.^58^ and Stukalov et al.^59^) and viral RNA-host protein interactions (e.g. Schmidt et al.^60^ and Flynnet al.^61^). Our data fusion framework is general and can be easily adapted to add these new types of interactions (viral RNA-host protein interactions) by extending the viral data type to also include viral RNA. Moreover, we want to highlight that we took a holistic approach and do not restrict the data to any tissue (e.g., lung tissue), since it has been shown that COVID-19 is a systemic disease with symptoms in multiple organs (e.g., lung, heart, kidneys and brain)^62^. Thus, our holistic approach allowed us to find drugs targeting NO signaling, which functions in different aforementioned tissues.

The framework we adapt in this study differs from other network-based computational studies for drug re-purposing applied to COVID-19 (such as Morselli Gysi et al.^19^; Sadegh et al.^18^) in the following: we do not only predict drugs to be re-purposed but also new candidate target genes. In particular, Morselli Gysi et al.^19^ ranked candidate drugs by aggregating the predictions of three different network-based methods: proximity, diffusion and AI network, based on their efficacy for COVID-19. The approach of Sadegh et al.^18^ is based on a group of seed nodes, which can be viral proteins and/or human genes, and then creating a subnetwork containing the seeds (using Steiner Tree algorithm), as well as ranking the drugs targeting the seeds using a centrality measure (degree, closeness, betweenness, or TrustRank). In contrast, the framework we adapt in this study is based on the fusion of several data sources, including chemical similarity of the drugs. Furthermore, the molecular interaction network that we generated for the host offers a more complete representation of the cell, as it includes information from several systems-level molecular interaction networks (protein–protein, genetic and metabolic interactions)^21^, whereas Morselli Gysi et al.^19^ and Sadegh et al.^18^ based its host molecular interactome only on the PPI network. We numerically compared our study to Morselli Gysi et al. by computing the overlap between the 149 drugs involved in our “common neighbor DTIs” list and the two lists they provided: top 100 drugs computationally predicted and 77 drugs experimentally validated (note that the overlap between these two sets of drugs provided by Morselli Gysi et al. is 9 drugs ($5.36\%$)). Thus, out of 149 drugs in our “common neighbor DTIs” list, 5 are in the computationally predicted list ($3.36\%$ of overlap as a percentage of our 149 predicted drugs) and 10 are in the experimentally validated list ($6.71\%$ of overlap as a percentage of our 149 predicted drugs). To numerically compare our results to Sadegh et al., we used the list of 8 approved drugs provided by their platform when it is run with its default parameters, since they do not provide any list of drugs in the main manuscript, they only provide a few use cases. Out of the 149 drugs in our “common neighbor DTIs” list, 4 are in their list of the 8 approved drugs ($1.77\%$ of overlap as a percentage of our 149 predicted drugs), which account for half of their drugs. Thus, although the three studies are based on completely different methodologies and different data, as explained above, we find that some drugs that we predicted as putative for repurposing are also suggested in the other studies, which supports our results, especially the overlap with the wet lab validated drugs.

The presented data fusion framework exhibits robust performance, as exemplified by its capability to identify previously predicted DTIs involving drugs under current clinical investigation. Beyond its application in this work, the framework is highly versatile and has been successfully applied to identify of cancer driver genes, patient stratification and drug re-purposing^21^. To exploit further this flexibility in the context of viral infections, the framework could be extended to search for the existing drugs with broad-spectrum antiviral activities by including information about host proteins targeted by more than one virus^63,64^. A recent example of such re-purposing is Remdesivir, developed initially against the hepatitis C virus and currently investigated as potential COVID-19 treatment^7^. Besides being economically more efficient, broad spectrum antivirals are by definition likely to act on commonly exploited host pathways that tend to be indispensable for viral replication. Thus, targeting such pathways will pose a higher evolutionary hurdle for the formation of viral resistance, which may circumvent the problems faced when designing highly virus-specific drugs^65^.

## Methods

### Datasets, pre-processing and matrix construction

We obtained the protein–protein interaction (PPI), genetic interaction (GI) and virus-host interaction (VHI) networks from the BioGRID database (version 3.5.183)^66^. VHIs were based on the dataset reported by Gordon et al.^5^, with $n_1 = 26$ viral proteins interacting with 332 host genes. We constructed the human PPI network with all physical interactions between human proteins reported by at least one of the following experiments: Two-hybrid, Affinity Capture-Luminescence, Affinity Capture-Western, Affinity Capture-MS; this resulted in 16,431 proteins (nodes) connected by 272,232 interactions (edges). We constructed the GI network with all the genetic interactions reported in BioGRID; this resulted in 3302 genes connected by 8333 interactions. We merged these two networks with the metabolic interaction (MI) network from the KEGG database (accessed in May 2020)^67,68^. We constructed the MI network by connecting all the genes that participate in the same metabolic pathway. In particular, we retrieved as metabolic pathways all the pathways in KEGG that contain at least one of the following keywords: metabolism, metabolic, glycolysis, TCA, oxidative phosphorylation, fatty acid, pentose, degradation or biosynthesis; this resulted in 1530 genes connected by 56,564 interactions. The resulting network from merging the PPI, GI and MI networks comprised 336, 159 interactions among $n_2 = 16,872$ genes. We termed this network the Molecular Interaction Network (MIN) (see Supplementary Fig. S1A,B for the overlap of genes and interactions of the three networks). Due to the small number of the host proteins interacting with the viral proteins (332 out of the 16, 872), the relational matrix, $R_{12}^{n_1 \times n_2}$, containing VHIs is highly sparse. Following our previous data fusion framework^21^, we applied a pre-processing step based on network propagation to smoothen this matrix. The procedure consisted of iteratively updating the $R_{12}^{n_1 \times n_2}$ using the following update rule: $R_{12}^{t+1} = \alpha R_{12}^{t}\overline{A_2} + (1-\alpha ) R_{12}^{0}$ where $\overline{A_2}$ is the normalized adjacency matrix of the MIN network computed as $\overline{A_2} = A_2D_2^{-1}$, $R_{12}^0$ is the initial $R_{12}$ and $\alpha$ is a tuning parameter that controls the distance of diffusion through the MIN network. We used $\alpha = 0.6$ and $| R_{12}^{t+1} - R_{12}^{t}| < 10^{-6}$ as convergence criterion to obtain the final network-smoothed matrix, $\overline{R_{12}^{n_1 \times n_2}}$.

We obtained the data related to the drugs from the DrugBank database (version 5.1.3)^69^. Drug-Target Interactions (DTIs) between the retrieved $n_3=8279$ drugs (FDA-approved and experimental) and the $n_2 = 16,872$ genes in our MIN were captured by the relation matrix $R_{23}^{n_2 \times n_3}$. This matrix is quite sparse as the known DTIs involve only 4, 420 drugs targeting 2, 241 genes. We used the Simplified Molecular-Input Line-Entry System (SMILES) information of these $n_3$ drugs to create the Drug Chemical Similarity (DCS) network. First, we converted this simplified notation of the chemical structure to a binary vector in which each coordinate represents a particular substructure from the set of all known sub-structures. Then, we computed the chemical similarity between two drugs based on the similarity between their vectors using Tanimoto similarity coefficient^70^. Once the similarity between all drug pairs is computed, we created a network containing the top $5\%$ most similar drug pairs, which resulted in 1, 727, 436 links.

### Data fusion framework tailored to SARS-CoV-2

We considered three different data types in our analyses: SARS-CoV-2 proteins, human genes and drugs and two relation types among them. SARS-CoV-2 proteins and human genes are related to each other by VHIs, which are captured in a smoothed high-dimensional relation matrix, $\overline{R_{12}^{n_1 \times n_2}}$, with $n_1$ viral proteins and $n_2$ human genes (for more details, see “Datasets, pre-processing and matrix construction” section); DTIs indicate relationships between human genes and drugs and are captured in a sparse high-dimensional binary relation matrix, $R_{23}^{n_2 \times n_3}$, for $n_2$ human genes and $n_3$ drugs, where its entries represent whether the product of a gene is targeted by a drug (1) or not (0). In addition to the relations among different data types, the relations between genes were captured by the MIN (for more details, see “Datasets, pre-processing and matrix construction” section), containing the known PPIs, GIs and MIs among them, whereas drugs relations were captured based on the similarity of their chemical structures, creating a DCS network. Both of these networks were represented by their Laplacian matrix, *L*, computed as: $L = D - A$, where *A* is the adjacency matrix and *D* is the diagonal degree matrix (i.e., whose entries on the diagonal are row sums of *A* and all other entries in *D* are zeros). Thus, $L_2^{n_2 \times n_2}$ and $L_3^{n_3 \times n_3}$ represent the MIN and DCS Laplacians, respectively. Figure 1a shows a schematic illustration of the datasets used in this study.

Following our previous data fusion methodology^21^, we used Graph-regularized non-negative matrix tri-factorization (GNMTF) to simultaneously decompose each of the two relation matrices into a product of three non-negative low-dimensional matrices while preserving the network structure of the MIN and DCS. The two decompositions, $R_{12} \approx G_1 H_{12} G_2^\top$ and $R_{23} \approx G_2 H_{23} G_3^\top$, share the matrix factor $G_2$ fusing the data via simultaneously decomposing the VHI and DTI networks. The network structure of the MIN and DCS is preserved by adding two regularization terms ($tr(G_2^\top L_2 G_2)$ and $tr(G_3^\top L_3 G_3)$, respectively), so that $G_2$ favors grouping together genes that interact in the MIN and that $G_3$ favors grouping together drugs that are chemically similar in the DCS network. Figure 1b shows an illustration of the GNMTF. Briefly, the low dimensional matrices can be obtained by solving the optimization problem shown in Eq. (1):

1 $$\begin{aligned} \min _{\genfrac{}{}{0.0pt}2{G_i \ge 0}{(1 \le i \le 3)}} J = \min _{\genfrac{}{}{0.0pt}2{G_i \ge 0 }{(1 \le i \le 3)}} \Big (||R_{12} - G_1 H_{12} G_2^\top ||^2_F + ||R_{23} - G_2 H_{23} G_3^\top ||^2_F + tr(G_2^\top L_2 G_2) + tr(G_3^\top L_3 G_3) \Big ), \end{aligned}$$

where $||\cdot ||_F$ denotes the Frobenius norm and *tr* denotes the trace of a matrix. The objective function, *J*, is heuristically minimized with an iterative method, starting from an initial solution and using multiplicative update rules to converge towards a locally optimal solution^71^. The final decomposition (used for predicting novel DTIs) was obtained by using the Singular Value Decomposition (SVD) as an initial solution and $\frac{|J_{n+1} - J_n|}{|J_n|} < 10^{-5}$ as the convergence criterion.

### Choosing the number of clusters

The number of clusters, $k_1$, $k_2$ and $k_3$, are key parameters of the GNMTF. However, there is no gold standard procedure to find a suitable values of these *k*’s. We used the procedure inspired by Brunet et al.^72^, consisting of choosing the parameter based on its cluster stability measured by the dispersion coefficient. In particular, the hard clustering procedure was applied to the corresponding matrix factor $G_i$, obtaining a clustering encoded in a connectivity matrix $C_i$, which is defined as a binary matrix where its rows and columns are the clustered entities (viral proteins, human genes or drugs) and 1 means that both entities belong to the same cluster. By applying this procedure with Random Acol initialization, we computed the average of the obtained $C_i$’s, $\overline{C_i}$, and measured the stability of these clusterings according to the dispersion coefficient: $\rho _{k_i} = \frac{1}{n^2} \sum _{l=1}^n \sum _{j=1}^n 4(C_{lj} - \frac{1}{2})^{2}$. The idea is to choose the value of $k_1$, $k_2$ and $k_3$ such that the obtained clusters are the most stable, i.e. for which the mean of $\rho _{k_1}, \rho _{k_2}, \rho _{k_3}$, $mean_{\rho _{k_1},\rho _{k_2},\rho _{k_3}} = \frac{\rho _{k_1} + \rho _{k_2} + \rho _{k_3}}{3}$, is at its maximum.

The stability of the obtained clusters depends on the size of the cluster, smaller clusters will be more stable, but without much biological meaning, with the extreme case being when we obtain as many clusters as there are molecules. Thus, we decided to focus the grid search around the rule of thumb, $k_i^{RT} = \sqrt{\frac{n_i}{2}}$, which is a heuristic to determine a fair number of clusters given the number of points $n_i$ that we need to cluster^73^. According to this heuristic, the number of clusters for each dataset is $k_1^{RT} \approx 3$, $k_2^{RT} \approx 90$, and $k_3^{RT} \approx 60$, corresponding to $n_1 = 26$ viral proteins, $n_2 = 16,872$ human genes and $n_3 = 8,279$ drugs. Therefore, we performed a grid search for the following values: $k_1 \in \{3,5\}$, $k_2 \in \{60,80,100,120\}$ and $k_3 \in \{40,60,80\}$. The most stable clustering was achieved by $k_1 = 3$, $k_2 = 120$ and $k_3= 80$ ($mean_{\rho _{k_1},\rho _{k_2},\rho _{k_3}} = 0.661$), which are the values that we used for the presented results (Supplementary Fig. S4).

### Extracting clusters of genes and drugs

The matrix factors $G_2^{n_2 \times k_2}$ and $G_3^{n_3 \times k_3}$, from GNMTF decomposition, are the cluster indicators of genes and drugs, respectively; based on their entries, $n_2$ genes are assigned to $k_2$ clusters and $n_3$ drugs are assigned to $k_3$ clusters, respectively. In particular, the hard clustering procedure of Brunet et al.^72^, was used to cluster the genes of the matrix factor $G_2^{n_2 \times k_2}$. The columns of $G_2^{n_2 \times k_2}$ correspond to the $k_2$ clusters and each gene is assigned to the cluster that has the largest entry in the gene’s row. The clusters can be represented by a binary connectivity matrix, $C_2^{n_2 \times n_2}$, where its rows and columns are the genes and 1 means that both genes belong to the same cluster. Similarly, we clustered the drugs of the matrix factor $G_3^{n_3 \times k_3}$ obtaining a connectivity matrix $C_3^{n_3 \times n_3}$ representing the clusters of drugs.

### Enrichment analysis of gene and drug clusters

To compute the functional enrichments of the common neighbor genes, either for the whole list of genes, or for the 49 common neighbor genes that were predicted to be targeted by FDA-approved drugs, we used the gprofiler Python package v.1.0.0 (parameters: organism=“hsapiens” source=c(“GO”,“KEGG”,“REAC”,“CORUM”))^74^. The p-value are corrected by the Set Counts and Sizes correction method^74^. This method considers the dependency of multiple tests by taking into account the overlap of functional term. We used this software for its capability to perform the enrichment analysis across multiple functional annotation databases.

To assess the quality of the obtained clusters of genes and drugs, we computed the enrichment of biological annotations in the clusters. For each gene (or equivalently, protein, as a gene product) in the network, we used the most specific experimentally validated Biological Process (BP), Cellular Component (CC) and Molecular Function (MF) annotations present in the Gene Ontology (GO)^75^, while for each drug we used the “Drug Categories”(DC) from DrugBank^69^. The probability that an annotation is enriched in a cluster was computed by using a hypergeometric test, i.e., sampling without replacement strategy shown in Eq. (2):

2 $$\begin{aligned} p = 1- \sum _{i=0}^{X-i} \frac{ \genfrac(){0.0pt}2{K}{i} \genfrac(){0.0pt}2{M-K}{N-i} }{\genfrac(){0.0pt}2{M}{N}}, \end{aligned}$$

where *N* is the number of annotated genes (drugs) in the cluster, *X* is the number of genes (drugs) in the cluster that are annotated with the given annotation, *M* is the number of annotated genes (drugs) in the network and *K* is the number of genes (drugs) in the network that are annotated with the annotation in question. Annotations with a Benjamini–Hochberg adjusted p-value^76^ of $p \le 0.05$ were considered to be statistically significantly enriched. We measured the quality of the clustering by computing three percentages: out of the total number of clusters of genes (drugs), the percentage that have GO terms (Drug Categories) enrichments; in all clusters of genes (drugs) taken together, the percentage of all leaf GO terms (Drug Categories) in them that are enriched in at least one cluster; and in all clusters of genes (drugs) taken together, the percentage of all genes (drugs) in them out of all human genes (drugs) in the network that have at least one of their annotations enriched in their clusters. To assess if an observed enrichment is greater than or equal to an enrichment by chance, we randomly shuffled (permutated) the values in the drug and gene matrix factors respectively and we used the permutation test: $p = \frac{r+1}{n+1}$, where *r* is the number of permutations that have an enrichment greater than or equal to the observed enrichment and $n = 100$ is the number of permutations that we used. We consider an enrichment to be statistically significant if the corresponding p-value is lower than or equal to 0.05.

### Prediction of new drug–target interactions for drug re-purposing

To capture new drug-target interactions, we exploited the matrix completion property of the GNMTF framework. This property consists of reconstructing the drug–target relational matrix from the obtained low-dimensional factors as $\widehat{R_{23}} = G_2 H_{23} G_3^\top$. Each entry of the reconstructed matrix contains an association score, $s_A$, corresponding to a drug–gene pair. This score can be interpreted as a relative measure of confidence for each drug–gene association. To assess that the score $s_A$ can be used to separate DTIs from non-interacting pairs performing precision-recall (PR) and receiver operating characteristic (ROC) curves analysis using all the input DTIs as ground truth. Then, to validate that that $s_A$ score can predict unseen DTIs by using tenfold cross-validation, we perform a tenfold cross-validation with stratified folds (i.e., ensuring the folds preserve the percentage of samples for each class). We used as ground truth the input DTIs (i.e., those DTIs present in DrugBank). Finally, to predict new DTIs, we define an optimal threshold based on $s_A$ score using F1-score and, then, we consider the false positive as predicted DTIs.

### Analysis of the molecular interaction network and its wiring patterns

To compute whether the overlap between the viral interactors (VIs) neighbor gene set and the differentially expressed genes (DEGs) neighbor gene set is significant, we performed a Hypergeometric Test (see Eq. (2)) where *N* is the number of genes that are the neighbors of VI genes, *X* is the number of genes that are both the neighbors of DEGs and the neighbors of VIs, *M* is the total number of genes in the network and *K* is the number of genes that are the neighbors of DEGs. Thus, *p* is the probability that the number of genes in the overlap is obtained by chance.

We analyzed the MIN using the following network properties: four centrality measures (degree, eigenvector, betweenness and closeness centrality) and the clustering coefficient (for more details, see Pržulj et al.^29^). The degree of a node is defined as the number of edges connected to the node and indicates the number of interactions in which the node is involved. The eigenvector centrality of a node is based on the importance of its neighbors, which is computed using the spectrum of the network and thus, identifies nodes connected to many highly connected nodes. The betweenness centrality of a node is the ratio of the number of shortest paths from all vertices to all others that pass through the node over all shortest paths and thus, nodes with high betweenness centrality are bottlenecks in the network, meaning that these nodes are more crucial in linking dense regions of the network. The closeness centrality quantifies how close a node is to all other nodes by computing the average of the lengths of the shortest paths from the node to all other nodes in the network. The clustering coefficient is the fraction of triangles that touch the node over all possible triangles in its neighborhood of the node and it captures whether the neighbors of a given node tend to cluster. We used these statistics to compare the relevant sets of genes for COVID-19 (VI, DEG, VI-unique neighbors, DEG-unique neighbors, common neighbors and background genes) and tested for statistically significant ($p < 0.05$) differences in the network statistics of these node sets by using a two-sided Mann–Whitney–Wilcoxon test.

The most sensitive measures capturing the local wiring patterns around nodes in networks are based on graphlets. Graphlets are defined as connected, non-isomorphic, induced subgraphs of large networks^31^. Different topological positions within graphlets are characterized by different symmetry groups of nodes, called automorphism orbits^77^. Orbits are used to generalize the notion of the node degree: the graphlet degrees of a node are the numbers of times a node is found at each orbit position. Yaveroǧlu et al.^32^ proved the existence of redundancies and dependencies between these orbits and proposed a set of 11 non-redundant orbits for 2- to 4-node graphlets (Supplementary Fig. S5). Thus, the wiring patterns of each node in the network can be represented by using the 11-dimensional vector, called Graphlet Degree Vector (GDV), or Graphlet Degree Vector Signature, which captures the 11 non-redundant graphlet degrees of a node^30^. To compare the wiring patterns of the different sets of nodes (VIs, DEGs, common and unique neighbors), we therefore calculated the GDV signature for each set of nodes and compared the average signatures of the different sets.

## Supplementary information

Supplementary Information 1.

Supplementary Information 2.

Supplementary Information 3.

Supplementary Information 4.

Supplementary Information 5.

Supplementary Information 6.

Supplementary Information 7.

Supplementary Information 8.

Supplementary Information 9.

Supplementary Information 10.

Supplementary Information 11.

Supplementary Information 12.

## Data Availability

Data reported in the paper are publicly available at https://gitlab.bsc.es/czambran/sweet-spot-for-therapeutic-intervention-for-covid-19.

## Code availibility

All the scripts used to generate the networks, integrate the data, perform the experiments, and analyze the data are coded in Python (v3.6.5) and require NumPy, Pandas, SciPy, SKLearn, NetworkX, MatplotLib, Matplotlib-venn, Statannot, Statsmodels, Gprofiler-official, and Rdkit libraries. The bar plots displaying results of enrichments analysis performed by Gprofiler were obtained using the enrichplot library from R.
