# Supplementary material for: Network neighbors of viral targets and differentially expressed genes in COVID-19 are drug target candidates
Source: Sci Rep. 2021 Sep 23;11:18985. doi: 10.1038/s41598-021-98289-x (PMC8460804; doi:10.1038/s41598-021-98289-x)
Supplement: Supplementary file 12 — Supplementary Information 12. [file 41598_2021_98289_MOESM12_ESM.pdf]

Supplementary Information for: Network neighbors of viral targets and differentially expressed genes in COVID-19 are drug target candidates

Carme Zambrana[1], Alexandros Xenos[1], Noël Malod-Dognin[1,2], René Böttcher[1], and Nataša Pržulj[1,2,3,*]

[1]Barcelona Supercomputing Center, Barcelona, Spain.
[2]Department of Computer Science, University College London, London, WC1E 6BT, United Kingdom.
[3]ICREA, Pg. Lluís Companys 23, Barcelona, Spain.
[*]natasha@bsc.es

Supplementary Results

The structure of PPI is preserved when merging it with GI and MI.

To validate that after merging the three networks the structure of the PPI network is preserved, we analyze the MIN and the constituent networks, PPI, GI and MI, by the following commonly used network properties: four centrality measures (degree, eigenvector, betweenness and closeness centrality) and clustering coefficient (for more details, see section "Analysis of the molecular interaction network and its wiring patterns" in Methods). As shown in Supplementary Table S1, MI has highest values in all the network properties, especially for the clustering coefficient, which is expected since the MI connects all the genes that participate in the same metabolic pathway. The MIN has similar values to those for PPI network, which is expected since the PPI network is the biggest one compared to the other constituent networks, GI and MI (see Supplementary Figure S1A-B). Thus, in terms of centrality and clustering, the PPI network structure is preserved when merging it with the GI and MI networks.

To assess whether the wiring patterns of the PPI are preserved in the MIN, we use Graphlet Degree Vectors (GDVs) to compare the MIN and the three constituent networks, PPI, GI and MI (for more details, see section "Analysis of the molecular interaction network and its wiring patterns" in Methods). As shown in Supplementary Figure S1C, GDV of the MI network is very different from the GDV of the rest of the networks, especially in the clique-orbits. Namely, orbits 3, 9, 10, 12 and 14. This is expected because of how the MI network is constructed, (i.e., by connecting all the genes that participate in the same metabolic pathway). The GDV of the MIN is very similar to that of the PPI, showing that the wiring patterns on the PPI are preserved after the merging.

To specify the relation between the genes that the data fusion process must conserve, we construct the MIN by merging three different interaction networks: protein-protein interaction (PPI), genetic interaction (GI) and metabolic interaction (MI) networks. By doing so, we want to add to the PPI different types of relations between genes that are key for the SARS-CoV-2 infection, (e.g., we add the MI network since it has been demonstrated that metabolic processes, such as glycolysis, promote SARS-CoV-2 replication) without losing its structure.When comparing the network properties and GDV of the constituent networks and the MIN, we showed that the PPI structure is preserved in the MIN although the MI has a very different structure due to how it is constructed. Therefore, we obtain a holistic view of the relationship between genes without losing the PPI structure, which is the most curated of the constituent networks.

The data fusion framework preserves the biological relations between genes and drugs

After obtaining the factor matrices, we cluster the genes and drugs by applying hard clustering to the corresponding matrix factors, G_2 and G_3, respectively (for more details, see section "Extracting clusters of genes and drug" in Methods). As a validation step, we assess that the framework captures the functional relationships between genes (as captured by Gene Ontology (GO) annotations) and between the drugs (as captured by DrugBank "Drug Category" (DC) annotations), by performing an enrichment analysis on the gene and drug clusters obtained by the framework (for more details, see section "Enrichment analysis of gene and drug clusters" in Methods). As shown in Figure S2, more than 80% of the of clusters of genes have GO term enrichments for the three GO domains (i.e. Biological Process, Cellular Component, Molecular Function), while at least 15% of the genes have at least one of their annotations enriched in their clusters over all annotated genes. Similarly, 90% of the clusters of drugs have DC enrichments (Figure S2). To assess if an observed enrichment is greater than or equal to an enrichment that may be by chance, we perform a permutation test (for more details, see section "Enrichment analysis of gene and drug clusters" in Methods). The enrichments of both the gene and the drug clusters are statistically significant compared to

randomly generated clusters (p-value ≤ 0.01), confirming that the joint decomposition of VHIs and DTIs successfully extracts meaningful information from these data and is capable of predicting novel drug-target relations.

The data fusion framework can predict unseen DTIs

To validate that the s_A scores in the reconstructed matrix can predict unseen DTIs, we perform a 10-fold cross-validation with stratified folds (i.e., ensuring the folds preserve the percentage of samples for each class). We used as ground truth the input DTIs (i.e., those DTIs present in DrugBank). As shown in Figure S3, the PR-AUC and the ROC-AUC for the validation set are lower than for the training set as expected (Training: PR-AUC=0.694 ± 0.004 and ROC-AUC=0.996 ± 0.001; Validation: PR-AUC=0.332 ± 0.014, ROC-AUC=0.847 ± 0.015; AUCs reported as the mean and standard deviation with respect to the 10 folds). However, as shown in Figure S3b, the precision of the method is still high in the validation set for high thresholds. In fact, the chosen threshold ($s_A = 0.296$) obtains a high precision (precision > 0.8 for all the folds) over the validation set.

The holistic view of the human interactome uncovers additional DTIs

To assess which is the improvement when using the holistic view of the relationship between genes (i.e., MIN) instead of the PPI network, we applied the same framework only using the PPI network as the relation that must be conserved during the data fusion process.

After obtaining the factor matrices, we cluster the genes and drugs by applying hard clustering to the corresponding matrix factors, G_2 and G_3, respectively (for more details, see "Extracting clusters of genes and drug" in Methods). As a validation step, we assess that the framework captures the functional relationships between genes (as captured by Gene Ontology (GO) annotations) and between the drugs (as captured by DrugBank "Drug Category" (DC) annotations)y, by performing an enrichment analysis on the gene and drug clusters obtained by the framework (for more details, see "Enrichment analysis of gene and drug clusters" in Methods). As shown in Figure S4, more than 80% of the of clusters of genes have GO term enrichments for the three GO domain (i.e. Biological Process, Cellular Component, Molecular Function), while at least 15% of the genes have at least one of their annotations enriched in their clusters over all annotated genes. Similarly, 90% of the clusters of drugs have DC enrichments (Figure S4). These results are very similar to those obtained when using the MIN (Figure 2 in the main document). Thus, the fusion framework successfully captures meaningful information encoded in the network either using the MIN or PPI network as the input of the genes relation that must be conserved.

To predict new DTIs, we used the matrix completion property to reconstruct the DTI matrix. Each entry of the reconstructed matrix contains an association score, s_A, corresponding to a drug-gene pair. This score can be interpreted as a relative measure of confidence for each drug-gene association (for more details, see section "Prediction of new drug-target interactions for drug re-purposing" in Methods). Then, we assess that the score s_A can be used to separate DTIs from non-interacting pairs performing precision-recall (PR) and receiver operating characteristic (ROC) curves analysis using all the input DTIs as ground truth. As shown in Supplementary Figure S5, these PR and ROC curves are very similar when using MIN or PPI, having the same ROC-AUC ($\mathit{ROC\text{-}AUC} = 0.997$) and almost identical PR-AUC ($\mathit{PR\text{-}AUC}$ PPI $= 0.704$; $\mathit{PR\text{-}AUC}$ MIN $= 0.696$). In addition, we showed that s_A score can predict unseen DTIs by using 10-fold cross-validation. As shown in Figure S6, the PR-AUC and the ROC-AUC for the validation set are lower than for the training set as expected (Training: PR-AUC=0.698 ± 0.006 and ROC-AUC=0.996 ± 0.001; Validation: PR-AUC=0.326 ± 0.014, ROC-AUC=0.845 ± 0.009; AUCs reported as the mean and standard deviation with respect to the 10 folds). Finally, to predict new DTIs, we define an optimal threshold based on s_A using F1-score and, then, we consider the false positive as predicted DTIs. The best F1-score ($F_1 = 0.733$) is associated with a threshold of $s_A = 0.340$, yielding 533 newly predicted DTIs with 399 drugs targeting 131 genes (Supplementary Table S3).

The list of predicted DTIs using MIN and PPI, have an overlap of 500 DTIs (see Supplementary Figure S5), meaning that by using PPI only 61.43% of the DTIs predicted using the MIN were also predicted when using the PPI. Moreover, only 33 out of the 533 DTIs predicted by using the PPI were not predicted by using the MIN (23 targeted by FDA-approved drugs and 10 by experimental ones). In particular, these 33 DTIs have small association scores (i.e., they are at the bottom of the list). Therefore, we obtain more putative DTIs by enforcing that the framework preserved not only PPI between genes but also GI and MI.

Supplementary Figures

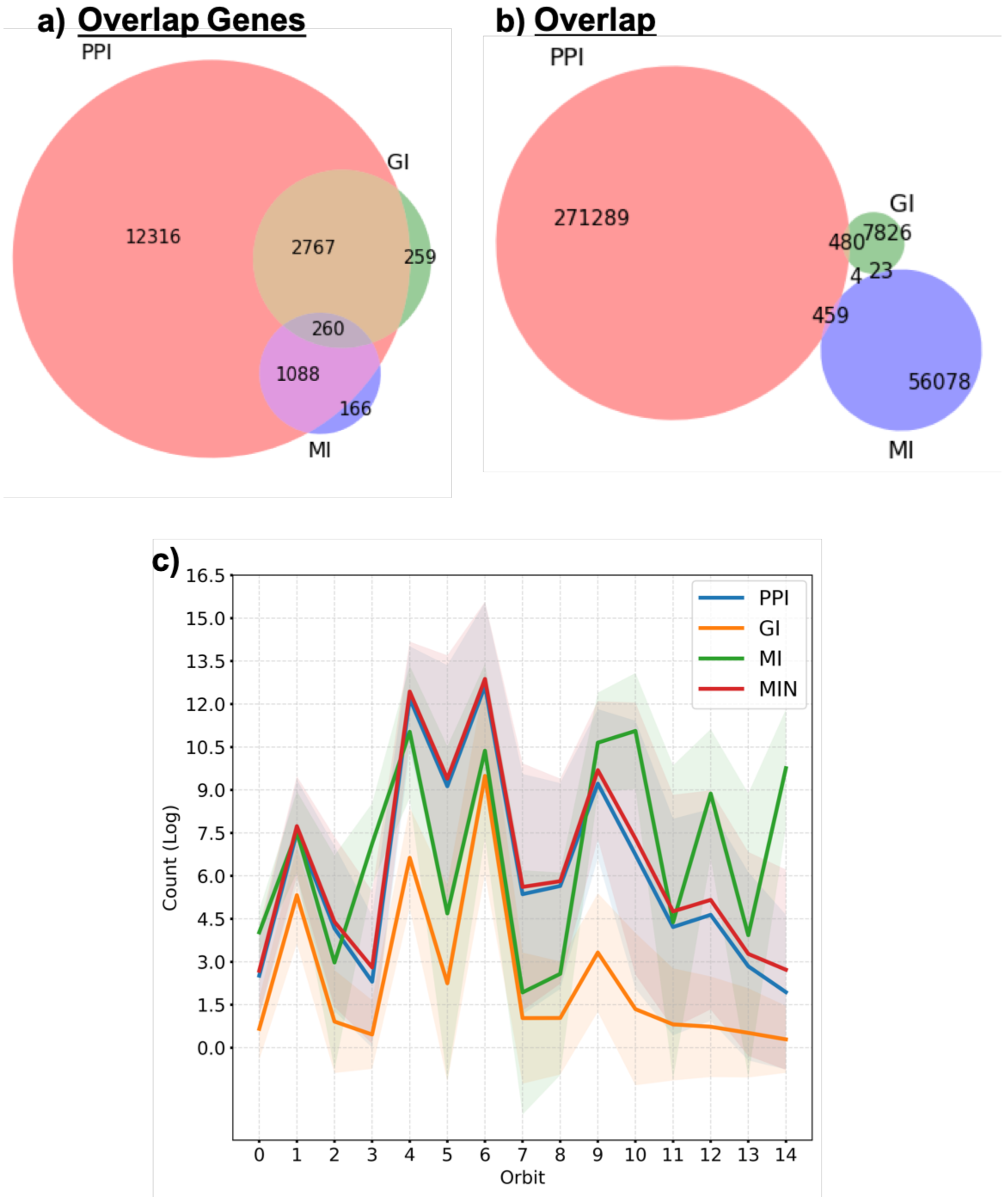

Supplementary Figure S1. Comparison between the molecular interaction network (MIN) and its constituent networks: protein-protein interactions (PPI), genetic interactions (GI) and metabolic interactions (MI) networks. **(a-b)** Overlap of the genes and interactions of the constituent networks, respectively. **(c)** GDV signature for the constituent networks and the MIN; counts (on the vertical axis) of the orbits (denoted by 0 to 14 on on the horizontal axis).

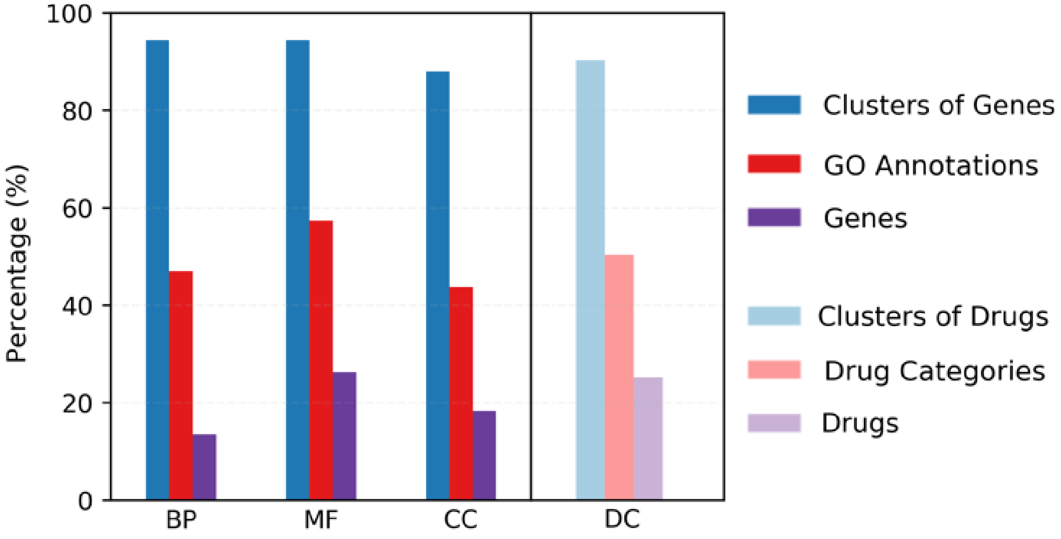

Supplementary Figure S2. Enrichment analysis for assessing the functional relevance of the gene and drug clusters obtained by the framework. The gene clusters are analyzed by using GO term annotations for the three domains: Biological Process (BP), Molecular Function (MF) and Cellular Component (CC); and the drug clusters are analyzed by using "Drug Categories" (DC) from DrugBank (horizontal axis). The probability that an annotation is enriched in a cluster was computed using a hypergeometric test. Then, we computed three percentages: out of the total number of clusters of genes (drugs), the percentage that have GO terms (Drug Categories) enrichments (in blue); in all clusters of genes (drugs) taken together, the percentage of all leaf GO terms (Drug Categories) in them that are enriched in at least one cluster (in red); and in all clusters of genes (drugs) taken together, the percentage of all genes (drugs) in them out of all human genes (drugs) in the network that have at least one of their annotations enriched in their clusters (in purple).

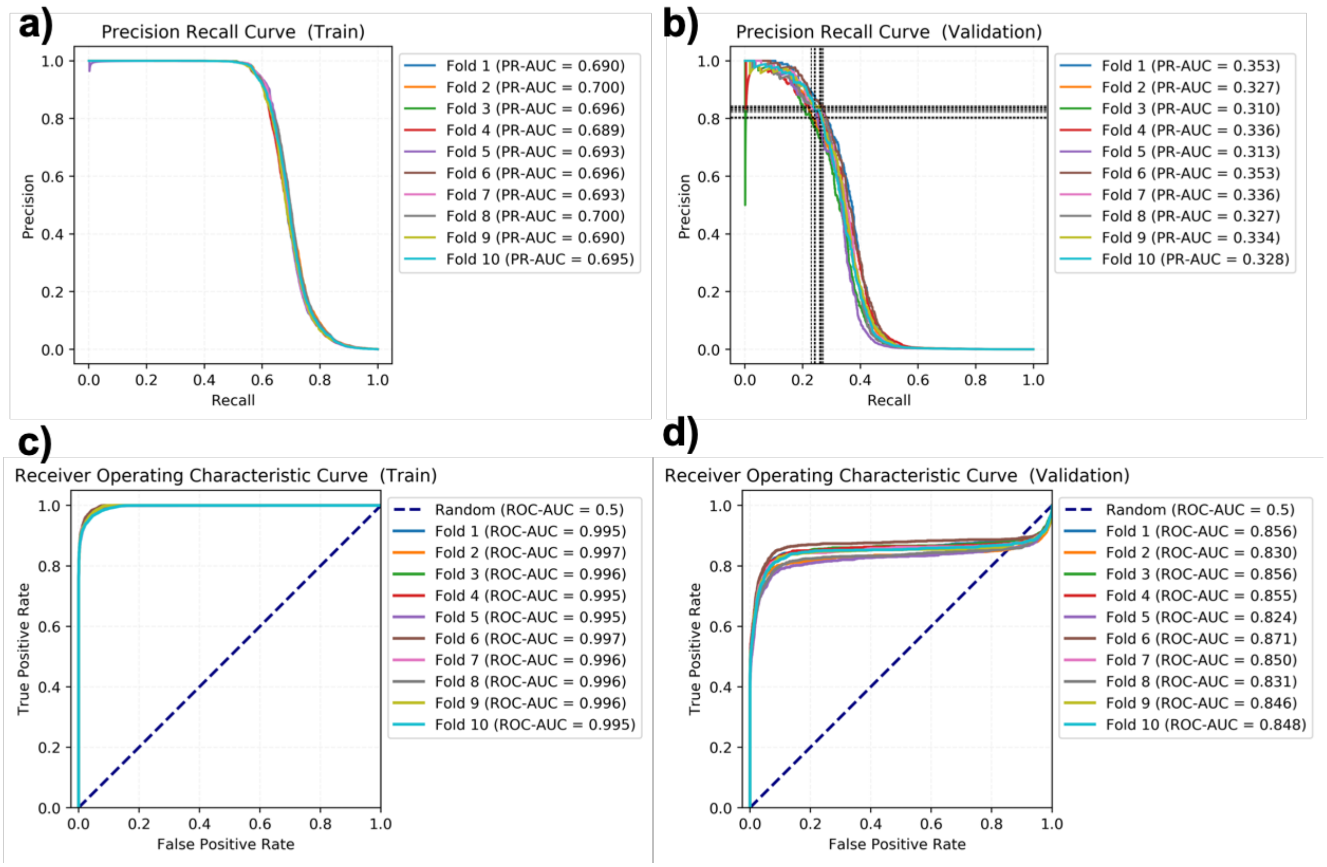

Supplementary Figure S3. Cross-validation assessing the predictive power of predicting new DTIs. We perform 10-fold cross-validation with stratified folds. **(a)** PR-curve over the training set (PR-AUC=0.694 ± 0.004), **(b)** PR-curve over the validation set (PR-AUC=0.332 ± 0.014), precision and recall obtained by the chosen threshold ($s_A = 0.296$) over the different folds are represented with dash lines, **(c)** ROC-curve over the training set (ROC-AUC=0.996 ± 0.001) and **(d)** ROC-curve over the validation set (ROC-AUC=0.847 ± 0.015). (The AUCs reported here are the mean and standard deviation with respect to the 10 folds).

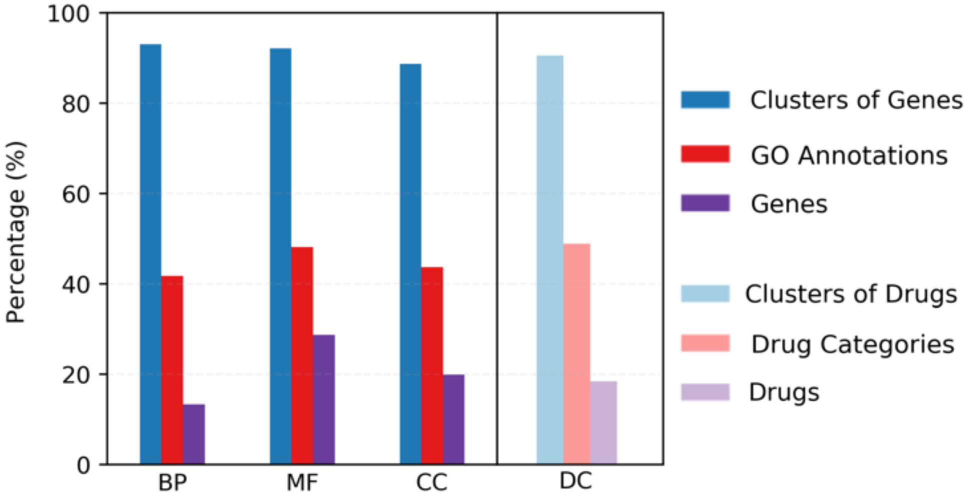

Supplementary Figure S4. Enrichment analysis for assessing the functional relevance of the gene and drug clusters obtained by the framework by only using the PPI. The gene clusters are analyzed by using GO term annotations for the three domains: Biological Process (BP), Molecular Function (MF) and Cellular Component (CC); and the drug clusters are analyzed by using "Drug Categories" (DC) from DrugBank (horizontal axis). The probability that an annotation is enriched in a cluster was computed using a hypergeometric test. Then, we computed three percentages: out of the total number of clusters of genes (drugs), the percentage that have GO terms (Drug Categories) enrichments (in blue); in all clusters of genes (drugs) taken together, the percentage of all leaf GO terms (Drug Categories) in them that are enriched in at least one cluster (in red); and in all clusters of genes (drugs) taken together, the percentage of all genes (drugs) in them out of all human genes (drugs) in the network that have at least one of their annotations enriched in their clusters (in purple) (in purple).

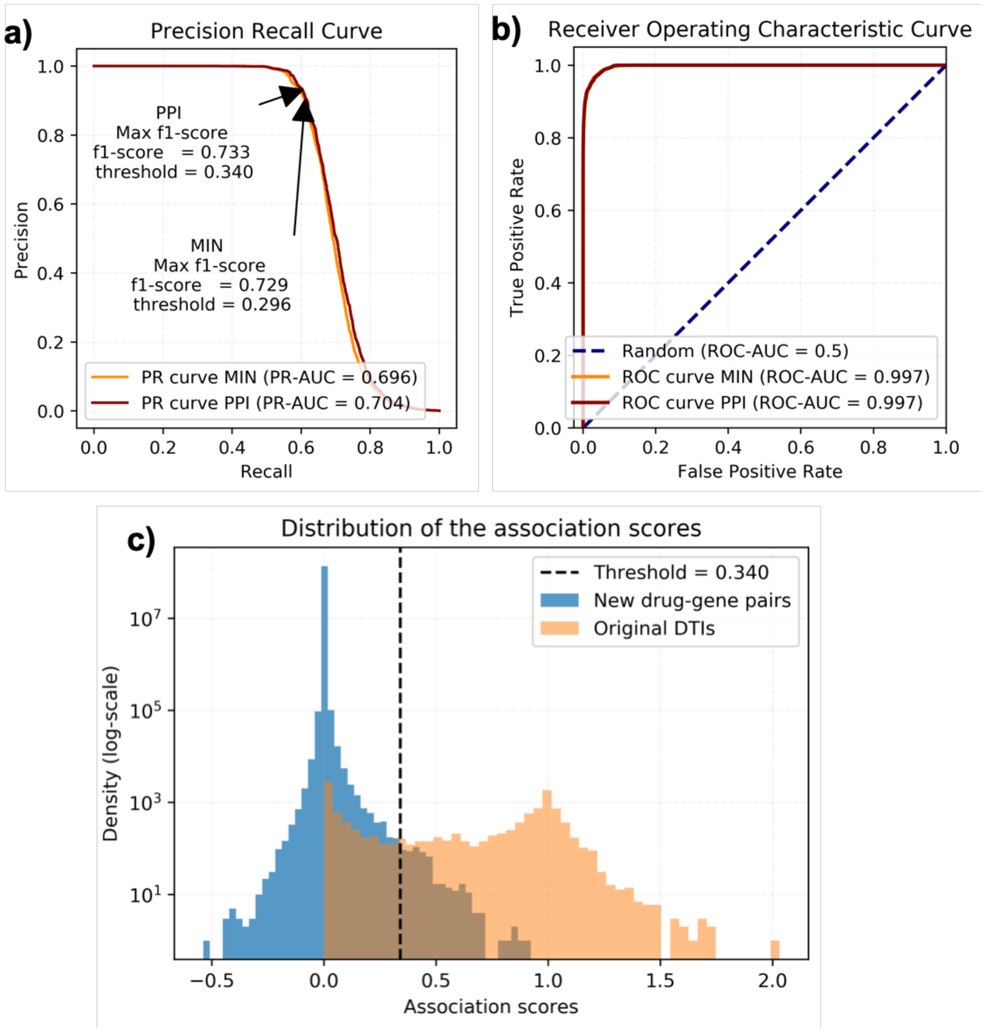

Supplementary Figure S5. **Prediction of new DTIs using only PPI as relation between genes.** **(a-b)** Comparison of the Precision-Recall (PR) and Receiver Operating Characteristic (ROC) curves for the framework using the MIN or only the PPI as the relation between genes. AUC - area under the curve. **(c)** Distribution of the association scores of the reconstructed matrix when only using the PPI as the relation between the genes; for the original DTIs (orange) and new drug-gene pairs obtained due to the matrix completion property of GNMTF (blue). New drug-gene pairs on the right side of the threshold (dashed line) were considered to be newly predicted DTIs.

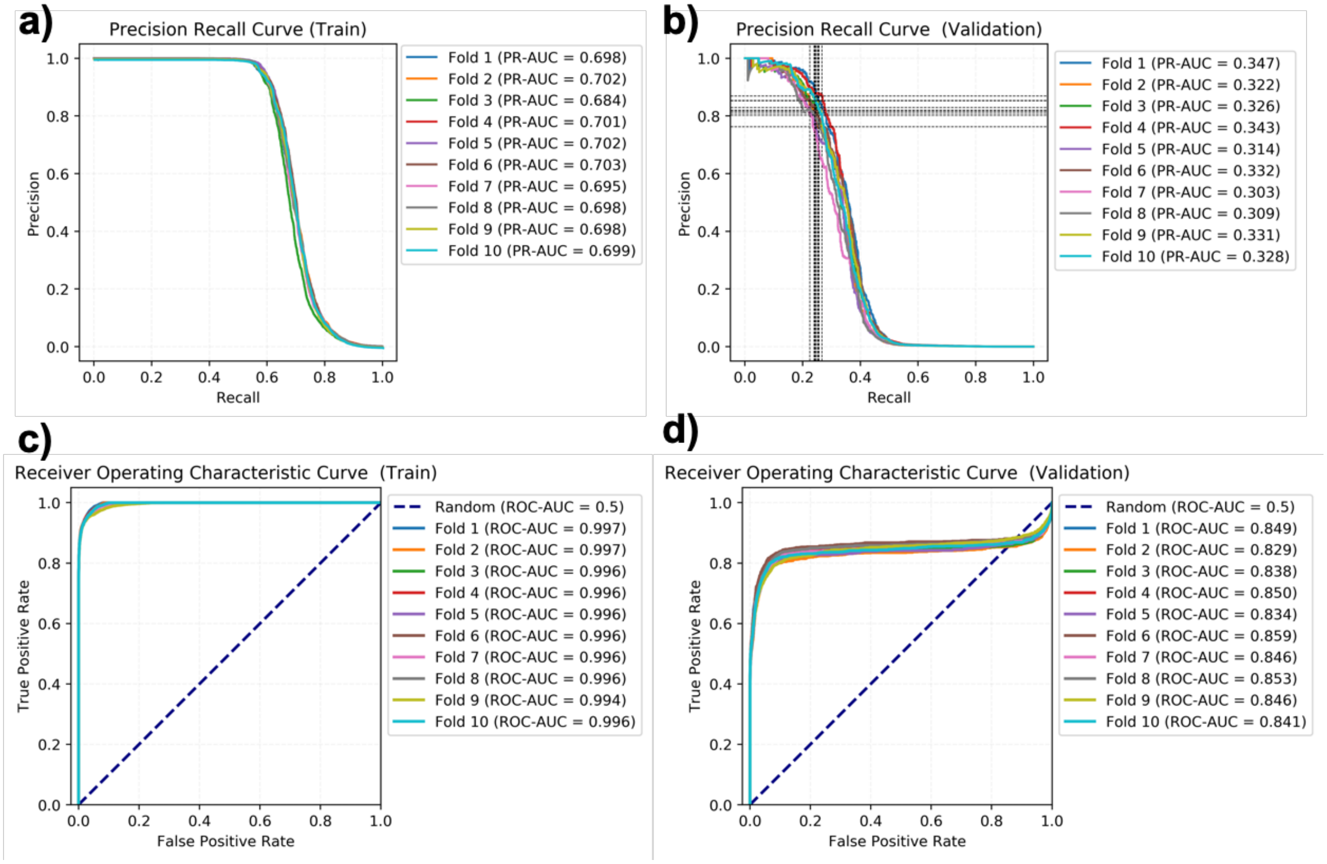

Supplementary Figure S6. **Cross-validation assessing the predictive power of predicting new DTIs.** We perform 10-fold cross-validation with stratified folds. **(a)** PR-curve over the training set (PR-AUC=0.698 ± 0.006), **(b)** PR-curve over the validation set (PR-AUC=0.326 ± 0.014), precision and recall obtained by the chosen threshold ($s_A = 0.340$) over the different folds are represented with dash lines, **(c)** ROC-curve over the training set (ROC-AUC=0.996 ± 0.001) and **(d)** ROC-curve over the validation set (ROC-AUC=0.845 ± 0.009). (The AUCs reported here are the mean and standard deviation with respect to the 10 folds).

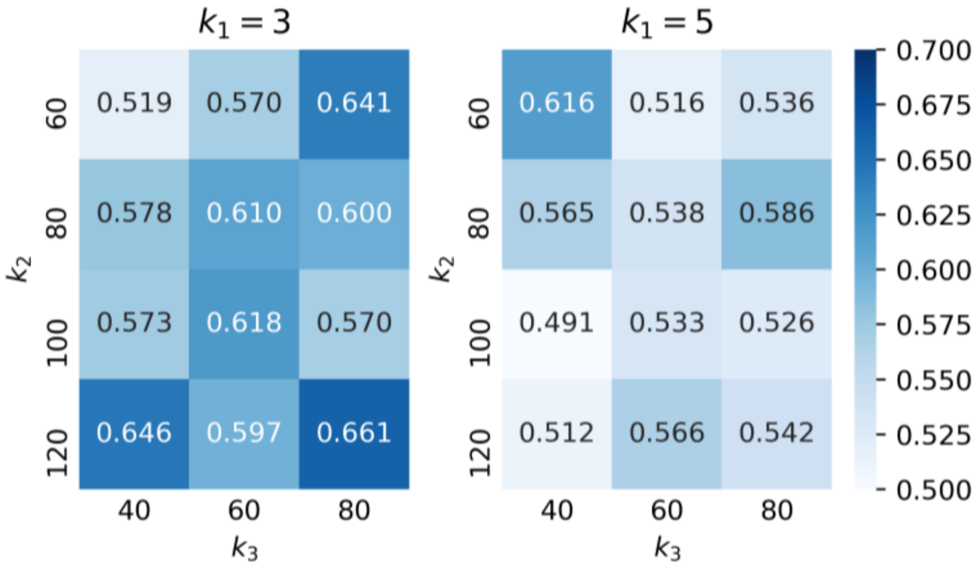

Supplementary Figure S7. Mean of the three dispersion coefficients (ρ_1. ρ_2. ρ_3) for all the values explored for choosing the parameters k_1. k_2 and k_3. Coefficients for $k_1 = 3$ are on the left and for $k_1 = 5$ on the right. The most stable clustering was achieved by $k_1 = 3$. $k_2 = 120$ and $k_3 = 80$ ($mean_{\rho_1.\rho_2.\rho_3} = 0.661$).

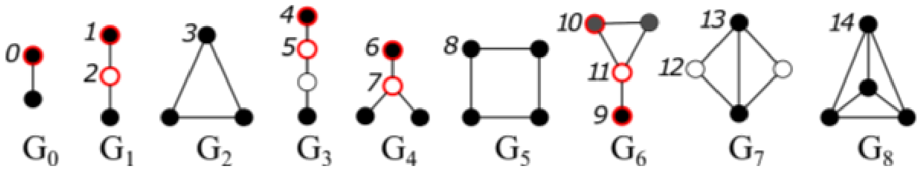

Supplementary Figure S8. Illustration of graphlets up to 4-nodes and their 15 automorphism orbits. The ten non-redundant orbits, whose counts cannot be derived from the counts of the other orbits. are highlighted in red.

Note: Supplementary Tables S2, S3, S4, S5, S6, S7, S8, S9, S10 and S11 are provided as comma-separated values (csv) files due to its extension.

Supplementary Table S1. Network properties of the molecular interaction network (MIN) and its constituent networks: protein-protein interaction (PPI), genetic interaction (GI) network and metabolic interaction (MI) network. The four networks are compared by the following commonly used network properties: four centrality measures (degree, eigenvector, betweenness and closeness centrality) and clustering coefficient. Note: this table is also provided as csv file.

	**Average Degree**	**Eigenvector Centrality**	**Clustering Coefficient**	**Betweeness Centrality**	**Closeness Centrality**
PPI	33.14	0.0032	0.1105	0.0001	0.3269
GI	5.05	0.0036	0.0623	0.0008	0.2552
MI	73.94	0.0137	0.8445	0.0011	0.344
MIN	39.85	0.0034	0.153	0.0001	0.3317

Supplementary Table S2. Predicted DTIs obtained by the data fusion framework. The list contains 814 newly predicted DTIs with 565 drugs targeting 172 genes. For each DTI the table contains the following information: the targeted gene (Gene); the drug involved in the DTI (DrugBank ID and Drug Name); association score (Score); DrugBank status of the drug, either FDA-approved or experimental (Drug Status); the gene set to which the targeted gene belongs, either VI, DEG, VI-unique neighbor, DEG-unique neighbor, common neighbor or background (Gene Description); and the databases in which the DTI was validated, either DrugCentral, CTD, TTD or PharmGKB (External Database).

Supplementary Table S3. Predicted DTIs obtained by the data fusion framework using only the PPI. The list contains 533 newly predicted DTIs with 399 drugs targeting 131 genes. For each DTI the table contains the following information: the targeted gene (Gene); the drug involved in the DTI (DrugBank ID and Drug Name); association score (Score); DrugBank status of the drug , either FDA-approved or experimental (Drug Status).

Supplementary Table S4. GDV counts (signature) comparison between the different gene sets. Each orbit (first column) is compared pair-wisely through all the gene sets (rest of the columns). We used the Mann-Whitney U test (with a significance level of 0.05) for each pair of orbits. The gene sets are: Viral interactors (VI), differentially expressed genes after infection (DEG), overlap of the direct network neighbors these two sets (common neighbors), neighbors of the VI and DEG gene set that were not in the common neighbors gene set (VI-unique neighbors and DEG-unique neighbors), and the rest of the genes in the MIN (background genes).

Supplementary Table S5. Functional enrichment analysis of the common neighbor genes using the gprofiler2 python-package. The table includes the adjusted p-values (P-value), the database and its domain (Source), the id and description of the term (Term id and Term name), number of genes associated with the term (Term size), number of the genes in the query gene set that are found in the database (Query size), as well as their intersection (Intersection size), precision and recall are defined as intersection_size/query_size and intersection_size/term_size, respectively.

Supplementary Table S6. Functional enrichment analysis of the VI-unique neighbors genes using the gprofiler2 python-package. The table includes the adjusted p-values (P-value), the database and its domain (Source), the id and description of the term (Term id and Term name), number of genes associated with the term (Term size), number of the genes in the query gene set that are found in the database (Query size), as well as their intersection (Intersection size), precision and recall are defined as intersection_size/query_size and intersection_size/term_size, respectively.

Supplementary Table S7. Functional enrichment analysis of the DEG-unique neighbor genes using the gprofiler2 python-package. The table includes the adjusted p-values (P-value), the database and its domain (Source), the id and description of the term (Term id and Term name), number of genes associated with the term (Term size), number of the genes in the query gene set that are found in the database (Query size), as well as their intersection (Intersection size), precision and recall are defined as intersection_size/query_size and intersection_size/term_size, respectively.

Supplementary Table S8. Functional enrichment analysis of the background genes using the gprofiler2 python-package. The table includes the adjusted p-values (P-value), the database and its domain (Source), the id and description of the term (Term id and Term name), number of genes associated with the term (Term size), number of the genes in the query gene set that are found in the database (Query size), as well as their intersection (Intersection size), precision and recall are defined as intersection_size/query_size and intersection_size/term_size, respectively.

Supplementary Table S9. List of the 5870 common neighbor genes in the MIN. The common neighbor genes are at the same time neighbors of the human proteins targeted by SARS-CoV-2 proteins (viral interactors (VI)) and neighbors of the genes that are differentially expressed after the infection (DEGs).

Supplementary Table S10. Predicted DTIs containing FDA-approved drugs targeting common neighbors (common neighbors DTIs). The list contains 185 newly predicted DTIs targeting 49 common neighbors with 149 drugs. For each DTI the table contains the following information: the targeted gene (Gene), the drug involved in the DTI (DrugBank ID and Drug Name), association score (Score), the databases in which the DTI was validated (External Database), whether the drugs were previously found in COVID-19 context (CORDITE) and number of interventional clinical trials for COVID-19 in which the drugs are currently studied (Clinical Trials).

Supplementary Table S11. Functional enrichment analysis of 49 genes involved in the 185 common neighbor DTIs using the gprofiler2 python-package. The table includes the adjusted p-values (P-value), the database and its domain (Source), the id and description of the term (Term id and Term name), number of genes associated with the term (Term size), number of the genes in the query gene set that are found in the database (Query size), as well as their intersection (Intersection size), precision and recall are defined as intersection_size/query_size and intersection_size/term_size, respectively.
